# ETMB-RBF: Discrimination of Metal-Binding Sites in Electron Transporters Based on RBF Networks with PSSM Profiles and Significant Amino Acid Pairs

**DOI:** 10.1371/journal.pone.0046572

**Published:** 2013-02-06

**Authors:** Yu-Yen Ou, Shu-An Chen, Sheng-Cheng Wu

**Affiliations:** Department of Computer Science and Engineering, Yuan Ze University, Chung-Li, Taiwan; Russian Academy of Sciences, Russian Federation

## Abstract

**Background:**

Cellular respiration is the process by which cells obtain energy from glucose and is a very important biological process in living cell. As cells do cellular respiration, they need a pathway to store and transport electrons, the electron transport chain. The function of the electron transport chain is to produce a trans-membrane proton electrochemical gradient as a result of oxidation–reduction reactions. In these oxidation–reduction reactions in electron transport chains, metal ions play very important role as electron donor and acceptor. For example, Fe ions are in complex I and complex II, and Cu ions are in complex IV. Therefore, to identify metal-binding sites in electron transporters is an important issue in helping biologists better understand the workings of the electron transport chain.

**Methods:**

We propose a method based on Position Specific Scoring Matrix (PSSM) profiles and significant amino acid pairs to identify metal-binding residues in electron transport proteins.

**Results:**

We have selected a non-redundant set of 55 metal-binding electron transport proteins as our dataset. The proposed method can predict metal-binding sites in electron transport proteins with an average 10-fold cross-validation accuracy of 93.2% and 93.1% for metal-binding cysteine and histidine, respectively. Compared with the general metal-binding predictor from A. Passerini et al., the proposed method can improve over 9% of sensitivity, and 14% specificity on the independent dataset in identifying metal-binding cysteines. The proposed method can also improve almost 76% sensitivity with same specificity in metal-binding histidine, and MCC is also improved from 0.28 to 0.88.

**Conclusions:**

We have developed a novel approach based on PSSM profiles and significant amino acid pairs for identifying metal-binding sites from electron transport proteins. The proposed approach achieved a significant improvement with independent test set of metal-binding electron transport proteins.

## Introduction

Cellular respiration is the process by which cells obtain energy from glucose. During respiration, cells break down simple food molecules, such as sugar, and release the energy they contain [1]. The point of cellular respiration is to harvest electrons from organic compounds such as glucose and use that energy to make a molecule called ATP (adenosine triphosphate). ATP in turn is used to provide energy for most cellular reactions.

**Figure 1:**
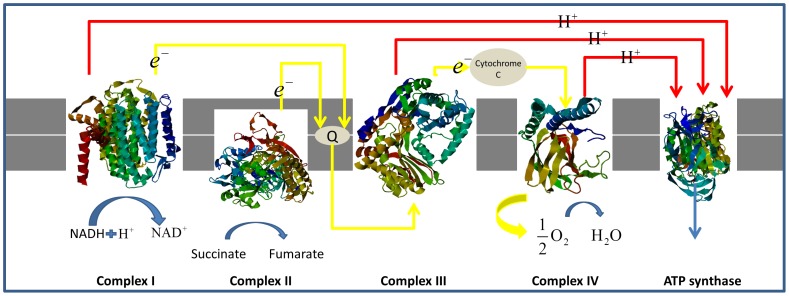
The electron transport chain in the inner membrane of mitochondria.

As cells do cellular respiration, they need a pathway to store and transport electrons, the electron transport chain. The function of the electron transport chain is to produce a trans-membrane proton electrochemical gradient as a result of oxidation-reduction reactions. If protons flow back through the membrane, ATP synthase converts this mechanical into chemical energy by producing ATP, which is provided energy in many cellular processes. The architecture of the electron transport chain with complex I–IV is given in Figure 1.

As Figure 1 shows, at the mitochondrial inner membrane, electrons from NADH and succinate pass through the electron transport chain to oxygen (Complex I(NADH dehydrogenase) and Complex II(succinate dehydrogenase)). Electrons pass from complex I to a carrier (coenzyme Q) embedded by itself in the membrane. From coenzyme Q electrons are passed to a Complex III (cytochrome b, c1 complex). Note that the path of electrons is from Complex I to Coenzyme Q to Complex III. Complex II, the succinate dehydrogenase complex, is a separate starting point, and is not a part of the NADH pathway. From Complex III the pathway is to cytochrome c then to a Complex IV (cytochrome oxidase complex). In the end, the proton electrochemical gradient allows ATP synthase to use the flow of H^+^ to generate ATP.

There are many oxidation-reduction reactions in the electron transport chain, such as NADH dehydrogenase, coenzyme Q – cytochrome c reductase, and succinate – coenzyme Q reductase. In these oxidation-reduction reactions in electron transport chains, metal ions play very important role as electron donor and acceptor. For example, Fe ions are in complex I and complex II, and Cu ions are in complex IV. Therefore, to identify metal-binding sites in electron transporters is an important issue in helping biologists better understand the workings of the electron transport chain. In this work, we try to develop a method based on Position Specific Scoring Matrix (PSSM) profiles and significant amino acid pairs to identify metal-binding residues in electron transport proteins.

In recent years, several methods have been proposed for predicting metal-binding sites (MBS) in proteins based on neural networks and support vector machines [2]–[5]. These work are major from A. Passerini and his co-workers except the work from Lin [2]. Prof. Passerini has proposed a two-stage machine-learning approach on their work [4]. The first stage consists of a support vector machine classifier, and the second stage consists of a bidirectional recurrent neural network. The authors of the work [4] have also published their web server as MetalDetector [5], which is the most popular web server for prediction metal-binding sites in proteins.

According to a recent comprehensive review 6], to establish a really useful statistical predictor for a protein system, we need to consider the following procedures: (i) construct or select a valid benchmark dataset to train and test the predictor; (ii)formulate the protein samples with an effective mathematical expression that can truly reflect their intrinsic correlation with the attribute to be predicted; (iii) introduce or develop a powerful engine to operate the prediction; (iv) properly perform cross-validation tests to objectively evaluate the anticipated accuracy of the predictor; (v) establish a user-friendly web-server for the predictor that is accessible to the public.

In this work, we propose a method based on PSSM profiles and significant amino acid pairs to identify metal-binding residues in electron transport proteins. We have selected a non-redundant set of 55 metal-binding electron transport proteins as our dataset. The proposed method can predict metal-binding sites in electron transport proteins with an average 10-fold cross-validation accuracy of 93.2% and 93.1% for metal-binding cysteine and histidine, respectively. Comparing with the general metal-binding predictor from A. Passerini et al., the proposed method can improve over 9% of sensitivity, and 14% specificity on the independent dataset in identifying metal-binding cysteines. The proposed method can also improve almost 76% sensitivity with same specificity in metal-binding histidine, and MCC is also improved from 0.28 to 0.88. The proposed approach achieved a significant improvement with independent test set of metal-binding electron transport proteins.

## Materials and Methods

**Figure 2:**
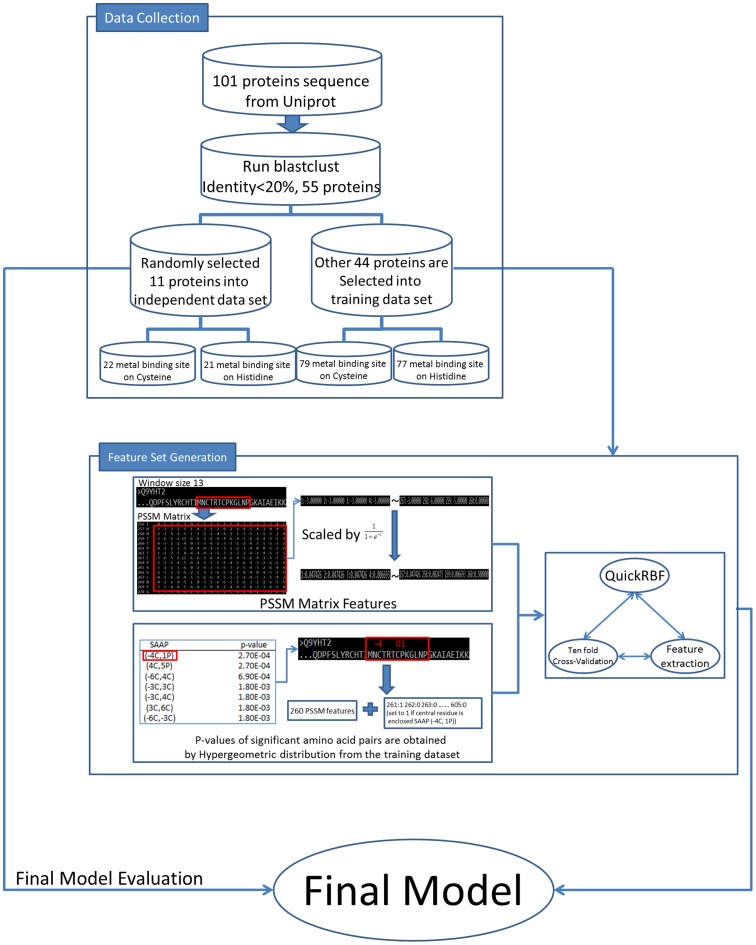
The flowchart of ETMB-RBF.

This work focuses on identifying metal-binding sites efficiently in electron transport proteins. As Figure 2 shows, the analyzing flowchart includes three sub-processes: data collection, feature set generation, and model evaluation. Following this model, we have developed a novel approach based on PSSM profiles and significant amino acid pairs for identifying metal-binding sites from electron transport proteins. The details of the proposed approach are described as follows.

### Data collection

First of all, we selected electron transport proteins with metal binding sites from UniProt database [7]. Then, we removed the sequences without the evidence at protein level and experimental metal-binding sites. Next, by using BLAST [8], the sequences with sequence identity more than 20% were excluded from the dataset. Since sequences falling below a 20% sequence identity can have very different structure [9], it is difficult to get a high success rate when tested by dataset in excluding homologous sequences with 20% sequence identity. Finally, 55 electron transport proteins are surveyed in this work.

The collected electron transport protein sequences were divided into two datasets: the training dataset and the independent test dataset. The training dataset is used for identifying metal binding sites and evaluating significant amino acid pairs in electron transport proteins. The training dataset includes 44 electron transport protein sequences which contain 79 metal-binding cysteine, 77 metal-binding histidine and 368 non-metal-binding cysteine and histidine. The independent test dataset, which includes 11 electron transport proteins which contain 22 metal-binding cysteine, 21 metal-binding histidine and 103 non-metal-binding cysteine and histidine, is used to evaluate the performance of the proposed method. The details of two datasets are listed in Table 1 and Table 2. Table 3 summarizes the statistics of structural topology and molecular function on 55 electron transporters in this work.

**Table 1:** The statistic of experimentally verified metal binding sites on electron transporters.

	Cross-Validation Dataset	Independent Test Dataset
Number of electron transporters	44	11
Number of metal binding cysteine	79	22
Number of metal binding histidine	77	21
Number of non-metal binding cysteine and histidine	368	103

**Table 2:** The catalytic of experimentally verified metal binding sites on electron transporters.

	Cross-Validation Dataset		Independent Test Dataset	
	Cysteine	Histidine	Cysteine	Histidine
Number of iron binding sites	72	63	18	15
Number of copper binding sites	7	14	4	6

**Table 3:** Details of electron transporters in the present study.

UniProt ID	Sequence length	Protein name	Num. of TM segment	Source	Molecular Function
Q5SJ79	562	Cytochrome c oxidase subunit 1	13	Thermus thermophilus	electron carrier activity;cytochrome-c oxidase activity;
P98002	558	Cytochrome c oxidase subunit 1-beta	12	Paracoccus denitrificans	electron carrier activity;cytochrome-c oxidase activity;
P0A405	755	PsaA	11	Thermosynechococcus elongatus	chlorophyll binding;
P51131	687	Cytochrome b/c1	10	Bradyrhizobium japonicum	electron carrier activity;oxidoreductase activity;
Q02761	445	Cytochrome b	8	Rhodobacter sphaeroides	Electron carrier activity;Oxidoreductase activity;
P32791	686	Ferric-chelate reductase 1	7	Saccharomyces cerevisiae	electron carrier activity;flavin adenine dinucleotide binding;
P0ABJ9	522	Cytochrome bd-I oxidase subunit I	7	Escherichia coli	electron carrier activity;oxidoreductase activity;
P06010	324	Reaction center protein M chain	5	Rhodopseudomonas viridis	electron transporter;
P0C0Y8	282	Reaction center protein L chain	5	Rhodobacter sphaeroides	electron transporter;
P0A444	360	Photosystem Q(B) protein 1	5	Thermosynechococcus elongatus	electron transporter;oxidoreductase activity;
P11695	311	Reaction center protein L chain	5	Chloroflexus aurantiacus	electron transporter;bacteriochlorophyll binding;
P17413	256	Fumarate reductase cytochrome b subunit	5	Wolinella succinogenes	oxidoreductase activity;
P11350	225	Cytochrome B-NR	5	Escherichia coli	electron carrier activity;nitrate reductase activity;
P00165	215	Cytochrome b6	4	Spinacia oleracea	electron transporter;oxidoreductase activity;
P0AEK7	217	FDH-N subunit gamma	4	Escherichia coli	electron carrier activity;formate dehydrogenase (NAD+) activity;
A5GZW8	159	CybS	3	Sus scrofa	ubiquinone binding;
D0VWV4	169	CYBL	3	Sus scrofa	electron carrier activity;succinate dehydrogenase activity;
P69054	129	Cytochrome b-556	3	Escherichia coli	electron carrier activity;succinate dehydrogenase activity;ubiquinone binding;
P0AC44	115	Succinate dehydrogenase hydrophobic membrane anchor subunit	3	Escherichia coli	electron carrier activity;succinate dehydrogenase activity;
P08306	298	Cytochrome c oxidase subunit 2	2	Paracoccus denitrificans	electron carrier activity;cytochrome-c oxidase activity;
P68530	227	Cytochrome c oxidase subunit 2	2	Bos taurus	electron carrier activity;cytochrome-c oxidase activity;
P00167	134	Cytochrome b5	1	Human	Aldo-keto reductase (NADP) activity;Cytochrome-c oxidase activity;
P95522	338	Apocytochrome f	1	Phormidium laminosum	electron carrier activity;
P00125	325	Cytochrome b-c1 complex subunit 4	1	Bos taurus	electron carrier activity;
Q02760	285	Cytochrome c1	1	Rhodobacter sphaeroides	electron carrier activity;
P04166	146	Cytochrome b5 type B	1	Rattus norvegicus	electron transporter;enzyme activator activity;
Q8DIP0	84	Cytochrome b559 subunit alpha	1	Thermosynechococcus elongatus	heme binding;
P95673	46	Light-harvesting protein B-800/850 beta 1 chain	1	Rhodospirillum molischianum	electron transporter;
Q8DIN9	45	Cytochrome b559 subunit beta	1	Thermosynechococcus elongatus	heme binding;
P07143	309	Cytochrome b-c1 complex subunit 4	1	Saccharomyces cerevisiae	electron transporter;
P0AAJ3	294	FDH-N subunit beta	1	Escherichia coli	electron carrier activity;formate dehydrogenase (NAD+) activity;
P20114	243	Cytochrome c1, heme protein	1	Euglena gracilis	electron carrier activity;
P08980	230	Rieske iron-sulfur protein	1	Spinacia oleracea	electron transporter;plastoquinol-plastocyanin reductase activity;ubiquinol-cytochrome-c reductase activity;
P49728	206	Rieske iron-sulfur protein	1	Chlamydomonas reinhardtii	plastoquinol-plastocyanin reductase activity;ubiquinol-cytochrome-c reductase activity;
P0CY48	191	Rieske iron-sulfur protein	1	Rhodobacter capsulatus	ubiquinol-cytochrome-c reductase activity;
P83794	179	Rieske iron-sulfur protein	1	Mastigocladus laminosus	electron transporter;plastoquinol-plastocyanin reductase activity;ubiquinol-cytochrome-c reductase activity;
Q5SJ80	168	Cytochrome c oxidase subunit 2	1	Thermus thermophilus	cytochrome-c oxidase activity;
P26789	53	Light-harvesting protein B-800/850 alpha chain	1	Rhodopseudomonas acidophila	electron transporter;bacteriochlorophyll binding;
P0C0Y1	49	Light-harvesting protein B-875 beta chain	1	Rhodobacter sphaeroides	electron transporter;bacteriochlorophyll binding;
P0A411	81	Photosystem I iron-sulfur center	Unknown	Anabaena variabilis	electron carrier activity;
Q7SIB8	102	Plastocyanin	Unknown	Dryopteris crassirhizoma	electron carrier activity;
P00289	168	Plastocyanin, chloroplastic	Unknown	Spinacia oleracea	electron carrier activity;
Q9YHT2	290	Iron-sulfur subunit of complex II	Unknown	Gallus gallus	electron carrier activity;succinate dehydrogenase activity;
P0A386	163	Cytochrome c-550	Unknown	Thermosynechococcus elongatus	electron carrier activity;
P09152	1247	Nitrate reductase A subunit alpha	Unknown	Escherichia coli	electron carrier activity;oxidoreductase activity;
Q56223	783	NADH-quinone oxidoreductase subunit 3	Unknown	Thermus thermophilus	electron carrier activity;NADH dehydrogenase (ubiquinone) activity;
P11349	512	Respiratory nitrate reductase 1 beta chain	Unknown	Escherichia coli	electron carrier activity;nitrate reductase activity;
P07173	356	Cytochrome c558/c559	Unknown	Rhodopseudomonas viridis	electron carrier activity;
P13272	274	Cytochrome b-c1 complex subunit 5	Unknown	Bos taurus	ubiquinol-cytochrome-c reductase activity;
P07014	238	Succinate dehydrogenase iron-sulfur subunit	Unknown	Escherichia coli	electron carrier activity;succinate dehydrogenase activity;
P27197	235	Auracyanin-B	Unknown	Chloroflexus aurantiacus	electron carrier activity;
Q8RMH6	162	Auracyanin-A	Unknown	Chloroflexus aurantiacus	electron carrier activity;
P18068	145	Plastocyanin, chloroplastic	Unknown	Chlamydomonas reinhardtii	electron carrier activity;
P82603	129	Cytochrome c-550	Unknown	Spirulina maxima	electron carrier activity;
Q56247	111	Cytochrome c-551	Unknown	Bacillus PS3	electron carrier activity;

### Feature set generation

#### Position Specific Scoring Matrix Profiles

In the structural point of view, several amino acid residues can be mutated without altering the structure of a protein, and it is possible that two proteins have similar structures with different amino acid compositions. Hence, the Position Specific Scoring Matrix (PSSM) profile is adopted, which have been widely used in protein secondary structure prediction, subcellular localization, classification of transporters, prediction of transport targets and other bioinformatics problems with significant improvement [10]–[20]. The PSSM profiles are obtained by using PSI-BLAST and non-redundant (NR) protein database.

PSSM profiles can be a useful feature set to represent evolutionary information in protein sequences [11], [21]. Life on Earth originated and then evolved from a common ancestor approximately 3.7 billion years ago, sequences are more similar among species that share a more recent common ancestor, and can be used to reconstruct evolutionary histories. In this work, we searched a very large sequences database (NR database) by using PSI-BLAST to find similar sequences of the target sequence. Then, we adopted the evolutionary information contained in PSSM profiles as input to radial basis function networks.

In the identification of metal binding sites on electron transport proteins, the generated PSSM profiles contained the probability of occurrence of each type of amino acid residues at each position. Each element in PSSM profile is scaled by 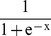 for normalizing the values between 0 and 1. The window size of 13 residues which the central residue is metal-binding site and 6 residues along both sides of the central residue is used for encapsulating an amino acid residue. Finally, 13 X 20 elements are used as PSSM feature set for identifying metal-binding sites. Features of non-metal-binding sites are generated by using the same approach as features of metal-binding sites.

In addition, we also generated different feature sets for comparison. There feature sets are generated by amino acid types(AA), BLOSUM62 matrix (BLOcks of Amino Acid SUbstitution Matrix) [22], and PAM250 matrix [23]. A matrix of 13 X 20 elements is used to represent each residue in a training dataset, where 13 denotes the window size and 20 elements from each row of the type of amino acids, BLOSUM62 matrix and PAM250 matrix.

#### Significant amino acid pairs

The significant amino acid pairs (SAAPs) around the metal-binding sites are identified based on the training dataset. These SAAPs are adopted to construct learning model for improving performance [24]. In order to make further investigations of substrate sites specificity, these SAAPs are identified based on statistical measurement of hypergeometric distribution. Each amino acid pairs surrounding metal-binding site is calculated p-value of hypergeometric distribution. The hypergeometric distribution is defined as:
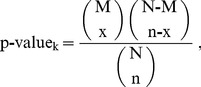
(1)where N denotes the number of sequences in the whole dataset, M denotes the number of sequences in the positive dataset, and (N-M) denotes the number of sequences in the negative dataset; n, x and n-x denotes the number of sequences which include the *k-*th SAAP in the whole dataset, in the positive dataset,and in the negative dataset respectively.

**Figure 3:**
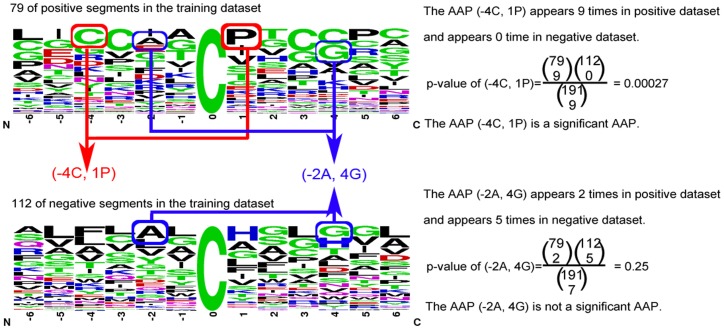
The illustration of calculating p-value for identifying significant amino acid pairs.

The amino acid pair surrounding metal-binding sites is significant when p-value is less than the significance level. It indicates that central residue is the metal-binding site with higher probability if significant amino acid pairs appear. As shown in Table 4, the most significant amino acid pair on cysteine is (−4C, 1P). (−4C, 1P), which suggests that the cysteine(C) on position −4 and the proline(P) on position +1 surrounding metal-binding sites is significant with p-value 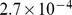. The illustration of calculating p-value for identifying significant amino acid pairs was shown in Figure 3.

**Table 4:** The significant amino acid pairs that surround the metal binding cysteine and histidine on electron transporters.

Metal binding cysteine		Metal binding histidine	
SAAP	p-value	SAAP	p-value
(−4C,1P)	2.70E-04	(−4C,−1C)	2.40E-06
(4C,5P)	2.70E-04	(−3C,2S)	6.00E-04
(−6C,4C)	6.90E-04	(−3V,4V)	6.00E-04
(−3C,3C)	1.80E-03	(−1C,1G)	7.20E-04
(−3C,4C)	1.80E-03	(1G,2S)	7.20E-04
(3C,6C)	1.80E-03	(−3A, −1C)	7.20E-04
(−6C, −3C)	1.80E-03	(−1C,4Y)	2.70E-03
(−6L,3C)	1.80E-03	(−1L,2F)	2.70E-03
(1I,6C)	4.50E-03	(1S,3D)	2.70E-03
(2G,6C)	4.50E-03	(−2A, −1C)	2.70E-03
(−2I,3C)	4.50E-03	(−2I,2F)	2.70E-03
(−4G,4C)	4.50E-03	(−2L,3M)	2.70E-03
(−5I, −3C)	4.50E-03	(−2P, −1C)	2.70E-03
(−6C, −4G)	4.50E-03	(−2V,1S)	2.70E-03
(−6C, −5I)	4.50E-03	(−3D, −2V)	2.70E-03
(−6C,5P)	4.50E-03	(−5F, −3C)	2.70E-03
(1I,3C)	8.60E-03	(−5V, −1C)	2.70E-03
(−3C, −2I)	8.60E-03	(−5V, −3A)	2.70E-03
(1P,3G)	1.10E-02	(−6E, −4C)	2.70E-03
(−5I,4C)	1.10E-02	(−2L, −1I)	2.80E-03
(−6G, −4Y)	1.10E-02	(−3L,5I)	2.80E-03
(−1G,3C)	1.90E-02	(−4C, −3A)	2.80E-03
(−3C,1H)	1.90E-02	(−5F,4G)	2.80E-03
(3C,4H)	1.90E-02	(−5F,5M)	2.80E-03
(−4G, −3C)	1.90E-02	(4G,5I)	7.60E-03

After calculating p-value for each amino acid pair surrounding metal-binding sites, the ranked SAAPs added into the feature set by using forward feature selection based on 10-fold cross-validation for improving predictive performance. Finally, 25 and 90 SAAPs are added into feature set of identifying metal binding cysteine and histidine, respectively. The final model was evaluated by using the independent dataset of 11 electron transporters.

The topmost 25 of SAAPs with p-value surrounding metal-binding cysteine and histidine are listed respectively in Table 4.

### Model evaluation

#### Design of the Radial Basis Function Networks

We have employed the QuickRBF package [25] to construct RBFN classifiers in this work. Also, the fixed bandwidth of 5 for each kernel function is employed in the network. In addition, we used all training data as centers. Then, the RBFN classifier identifies metal-binding sites based on the output function value. The details about network structure and design have been explained in our earlier article [26].

Classification based on radial basis function (RBF) networks has several applications in bioinformatics. It has been widely used to predict the cleavage sites in proteins [27], inter-residue contacts [28], protein disorder [29], the discrimination of β-barrel proteins [13], the classification of transporters [14], [16],the identification of O-linked glycosylation sites [24] , and the identification of ubiquitin conjugation sites [30].

The general mathematical form of the output nodes in an RBFN is as follows:
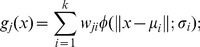
(2)

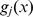
 is the function corresponding to the *j-th* output node and is a linear combination of *k* radial basis functions 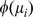 with center 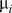 and bandwidth 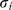; Also, 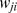 denotes the weight associated with the correlation between the j-th output node.

#### Assessment of predictive ability

The prediction performance was examined by 10-fold cross validation test, in which the training data were randomly divided into ten subsets of approximately equal size. The data were trained with nine subsets and the remaining set was used to test the performance of the method. This process was repeated 10 times so that every subset had been used as the test data once.

Sensitivity, specificity, accuracy, and MCC (Matthew's correlation coefficient) were used to measure the prediction performance. TP, FP, TN, FN are true positives, false positives, true negatives, and false negatives, respectively.
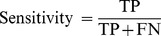
(3)

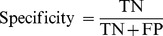
(4)

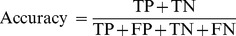
(5)




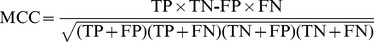
(6)


## Results and Discussion

### Predictive performance of metal-binding sites identification in electron transport proteins with different feature sets

We developed a variety of methods for metal-binding sites identification in electron transport proteins. The results obtained from the AA, BLOSUM62, PAM250, PSSM, and the combination of PSSM and SAAPs are presented in Table 5. The results showed that PSSM with SAAPs properties was successful in identifying metal-binding sites with an average 10-fold cross-validation accuracy of 93.2% and 93.1% for metal-binding cysteine and histidine, respectively. Our analysis showed that PSSM profiles and SAAPs properties had marginally improved the accuracy of identification, compared with the other feature sets.

**Table 5:** The ten-fold cross-validation performance of metal binding sites on Cross-Validation dataset.

ETMB-RBF with different features	True Positive	False Positive	True Negative	False Negative	Sensitivity	Precision	Specificity	Accuracy	MCC
Metal binding cysteine									
AA	60	22	90	19	75.9%	73.2%	80.4%	78.5%	0.56
BLOSUM62	65	12	100	14	82.3%	84.4%	89.3%	86.4%	0.72
PAM250	58	9	103	21	73.4%	86.8%	92.0%	84.3%	0.67
PSSM	76	16	96	3	96.2%	82.6%	85.7%	90.1%	0.81
PSSM+SAAPs	78	12	100	1	98.7%	86.7%	89.3%	93.2%	0.87
Metal binding histidine									
AA	37	30	226	40	48.1%	55.2%	88.3%	79.0%	0.38
BLOSUM62	43	21	235	34	55.8%	67.2%	91.8%	83.5%	0.51
PAM250	39	37	219	38	50.6%	51.3%	85.5%	77.5%	0.36
PSSM	60	13	243	17	77.9%	82.2%	94.9%	91.0%	0.74
PSSM+SAAPs	62	8	248	15	80.5%	88.6%	96.9%	93.1%	0.80

Combining the significant amino acid pairs with the sequence of amino acids increases the predictive accuracy specificity for metal-binding sites identification from 90.1% to 93.2% with metal-binding cysteine, and from 91.0% to 93.1% with metal-binding histidine. In addition, the sensitivity, precision specificity, and MCC are also improved. Consequently, according to the evaluation of 10-fold cross validation, the identified significant amino acid pairs can increase the predictive performance.

In statistical prediction, the following three cross-validation methods are often used to examine a prediction: independent dataset test, subsampling test, and jackknife test [6]. However, of the three test methods, the jackknife test is deemed the least arbitrary that can always yield a unique result for a given benchmark dataset. However, to reduce the computational time, we adopted the 10-fold cross validation and independent testing dataset test in this study.

### Comparison the performance with other method with independent test set

The independent test dataset, which includes 11 electron transport proteins which contain 22 metal-binding cysteine, 21 metal-binding histidine and 103 non-metal-binding cysteine and histidine, is used to evaluate the performance of the proposed method. As Table 6 shows, comparing with the general metal-binding predictor from A. Passerini et al., the proposed method can improve over 9% of sensitivity, and 14% specificity on the independent dataset in identifying metal-binding cysteines. The proposed method can also improve almost 76% sensitivity with same specificity in metal-binding histidine, and MCC is also improved from 0.28 to 0.88. This results shows that our method could be effectively used for indentifying metal-binding sites in electron transport proteins.

**Table 6:** Comparison performance with other methods.

Metal binding cysteine									
Method	True Positive	False Positive	True Negative	False Negative	Sensitivity	Precision	Specificity	Accuracy	MCC
Cross-Validation Dataset									
Metal Detector	78	26	86	1	98.7%	75.0%	76.8%	85.9%	0.75
ETMB-RBF	78	12	100	1	98.7%	86.7%	89.3%	93.2%	0.87
Independent Test Dataset									
Metal Detector	20	8	21	2	90.9%	71.4%	72.4%	80.4%	0.63
ETMB-RBF	22	4	25	0	100%	84.6%	86.2%	92.3%	0.85

Metal binding histidine									
Method	True Positive	False Positive	True Negative	False Negative	Sensitivity	Precision	Specificity	Accuracy	MCC
Cross-Validation Dataset									
Metal Detector	12	3	253	65	15.6%	80.0%	98.8%	79.6%	0.29
ETMB-RBF	62	8	248	15	80.5%	88.6%	96.9%	93.1%	0.80
Independent Test Dataset									
Metal Detector	2	0	74	19	9.5%	100%	100%	80.0%	0.28
ETMB-RBF	18	1	73	3	85.7%	94.7%	98.6%	95.8%	0.88

### The statistical analysis of amino acid compositions in electron transporters and general proteins

**Figure 4:**
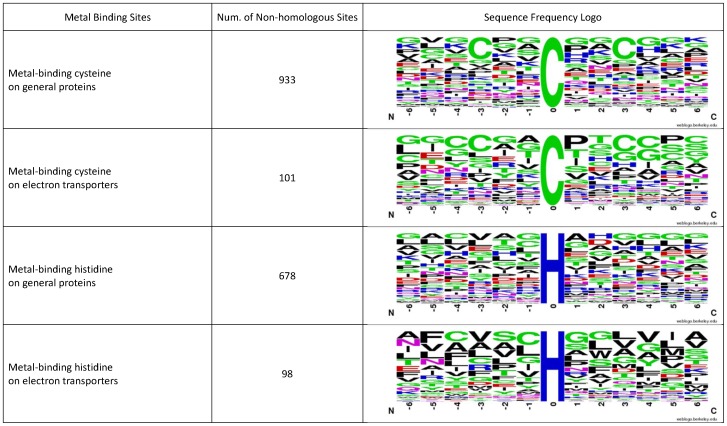
The sequence frequency logos of metal-binding cysteine and histidine in electron transporters and general proteins.

We have analyzed metal-binding cysteine and cysteine residues on electron transporters and general proteins. Using the sequences of electron transporters in Table 3, we generated the sequence logos of metal-binding cysteine and cysteine residues in electron transporters with flanking amino acids (−6 ∼ +6) by WebLogo [31], [32]. Also, we generated the sequence logos of metal-binding cysteine and cysteine residues in general proteins with the dataset in A. Passerini's work [4]. These four sequence logos are listed in Figure 4.

**Figure 5:**
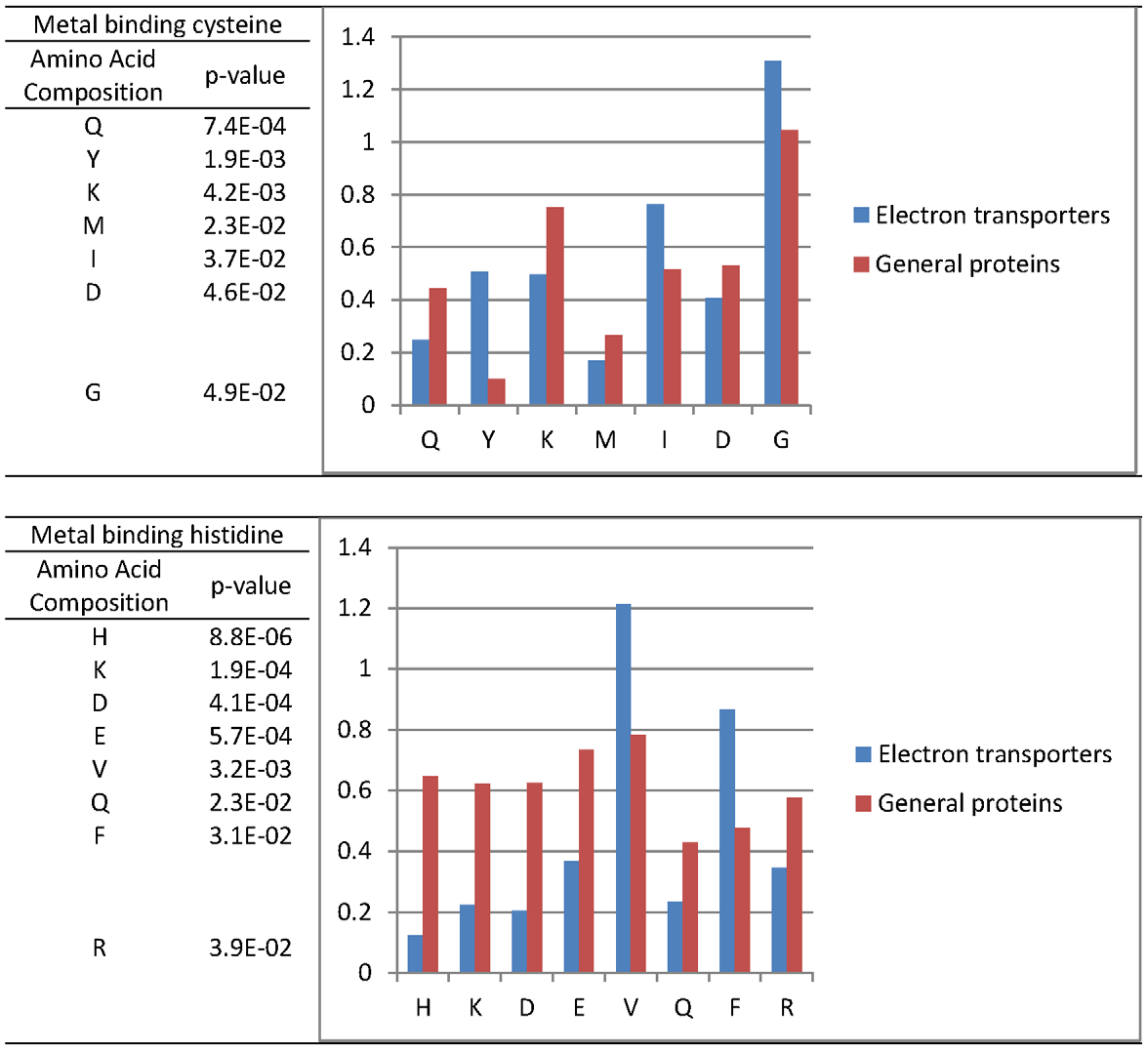
The significant amino acid compositions that surround the metal binding cysteine and histidine.

We also statistically analyzed the amino acid compositions with standard T-test of metal-binding cysteine and cysteine residues in electron transporters and general proteins. As Figure 5 shows, seven residues, Q, Y, K, M, I, D and G, surrounding metal-binding cysteine have significant difference between electron transporters and general proteins. Also, 8 residues, H, K, D, E, V, Q, F, and R, surrounding metal-binding histidine have significant difference between electron transporters and general proteins.

According the statistical analysis, the distribution of amino acids surrounding metal-binding residues are different between electron transport proteins and general proteins. This may be the reason why our proposed method performs better than the general metal-binding predictor.

## Conclusions

Cellular respiration is the process by which cells obtain energy from glucose, and is a very important biological process in living cell. As cells do cellular respiration, they need a pathway to store and transport electrons, the electron transport chain. The function of the electron transport chain is to produce a trans-membrane proton electrochemical gradient as a result of oxidation-reduction reactions. In these oxidation-reduction reactions in electron transport chains, metal ions play very important role as electron donor and acceptor. Therefore, to identify metal-binding sites in electron transporters is an important issue in helping biologists better understand the workings of the electron transport chain.

In this work, we proposed a method based on PSSM profiles and significant amino acid pairs to identify metal-binding residues in electron transport proteins. We have selected a non-redundant set of 55 metal-binding electron transport proteins as our dataset. The proposed method can predict metal-binding sites in electron transport proteins with an average 10-fold cross-validation accuracy of 93.2% and 93.1% for metal-binding cysteine and histidine, respectively. Comparing with the general metal-binding predictor from A. Passerini et al., the proposed method can improve over 9% of sensitivity, and 14% specificity on the independent dataset in identifying metal-binding cysteines. The proposed method can also improve almost 76% sensitivity with same specificity in metal-binding histidine, and MCC is also improved from 0.28 to 0.88. Our proposed approach achieved a significant improvement with independent test set of metal-binding electron transport proteins. The result shows that our method could be effectively used for indentifying metal-binding sites in electron transport proteins to help biologists better understand the workings of the electron transport chain.

Since user-friendly and publicly accessible web-servers represent the future direction for developing practically more useful models, simulated methods, or predictors, we will make efforts in our future work to provide a web-server for the method presented in this paper.
